# Co-Effects of Hydrological Conditions and Industrial Activities on the Distribution of Heavy Metal Pollution in Taipu River, China

**DOI:** 10.3390/ijerph191610116

**Published:** 2022-08-16

**Authors:** Qinglu Yao, Ling Chen, Lingchen Mao, Yu Ma, Fengyan Tian, Ruijie Wang, Xiang-Zhou Meng, Feipeng Li

**Affiliations:** 1 College of Environmental Science and Engineering, Tongji University, Shanghai 200092, China; 2 State Key Laboratory of Pollution Control and Resource Reuse, Tongji University, Shanghai 200092, China; 3 School of Environment and Architecture, University of Shanghai for Science and Technology, Shanghai 200093, China; 4 Shanghai Institute of Pollution Control and Ecological Security, Shanghai 200092, China; 5 Key Laboratory of Yangtze River Water Environment, Ministry of Education, College of Environmental Science and Engineering, Tongji University, 1239 Siping Road, Shanghai 200092, China

**Keywords:** Sediment, heavy metal, hydrodynamic, industrial activity, source apportionment

## Abstract

In Taipu River, after being transformed from a drainage channel to a drinking water supply river in 1995, heavy metals that have accumulated in sediments have become an environmental issue. Herein, we collected sediments of Taipu River in 2018, 2020, and 2021 and analyzed the distribution of Sb, As, Cd, Cu, Pb, Cr, and Zn to identify their sources. The results revealed that the mean concentrations of heavy metals were above the background values, except for Cr and As. During the non-flood season, the midstream of Taipu River becomes a heavy metal hotspot, with their concentrations 2–5 times higher than those in upstream sediment. There were significant correlations (r = 0.79–0.99) among drainage, precipitation and flow rate, which indicated that drainage caused by both the opening of Taipu Gate and precipitation control the flow rate and, then, possibly influenced the distribution of heavy metals. Moreover, three sources (industrial sources, particle deposition sources, and natural sources) were characterized as the determinants for the accumulation of heavy metal by the Positive Matrix Factorization model, with the contribution rates of 41.7%, 32.9%, and 25.4%, respectively. It is recommended that the influence of hydrological conditions and industrial activities should be a key consideration when developing regulations for the management of heavy metals in rivers.

## 1. Introduction

Despite the lethal effects of heavy metals, the uncontrolled usage of these metals in industries is still a problem. These excess heavy metals are discharged into the water through waste disposal, waste liquid discharge, and surface runoff, contaminating the water [1]. Sediments are generally considered to be an effective storage medium for heavy metals in rivers. These non-biodegradable heavy metals bioaccumulate in plants and animals, including humans, through the food chain and result in harmful effects [2]. In recent years, a number of studies focused on the typical heavy metal (e.g., Cr, Zn, Pb, Cu, Mn) contamination caused by a variety of industries in different environmental media [3,4,5], including Sb contamination by antimony deposits [6,7,8]. Nevertheless, little research has been conducted on Sb contamination caused by the textile industry, where Sb is often used as a flame retardant and catalyst [9,10].

Adsorption plays a crucial role in transferring heavy metals in the river to sediments on suspended particulate matter. At the same time, water flow has a considerable effect on the transport and deposition of suspended particulate matter and sediment [11]. The influence of hydrological conditions on the fate of heavy metals in sediments has been vastly studied in recent times. These studies suggested that hydrological conditions can control the physicochemical and redox conditions in the sediment, thereby affecting the transport and transformation of heavy metals [12,13,14]. Feng et al. [11] conducted a study on the downstream sediments of the Three Gorges Dam and concluded that upstream hydrological events (e.g., gate and dam construction, flooding) could have a significant impact on downstream areas. The hydrological conditions of estuaries and dams are of notable interest, but the complex hydrodynamics of plain river networks have rarely been studied. The heavy metals contamination in the plains of the river network, and their distribution by complex hydrological, developed industrial activities, etc., is becoming a prominent concern worldwide [11,15,16,17]. Many studies have found that the enrichment of heavy metals in sediments is closely related to the source of pollution [3,18,19]; however, the factors influencing the spatial and temporal distribution and enrichment of heavy metals remain unclear [20]. Adequate exploitation of the information contained in heavy metals can provide targets and directions for monitoring, prevention, and management of heavy metal pollution.

In order to improve the water quality in the Taihu Lake basin, the Taipu River was constructed in 1958, and the Taipu Gate was built to control the water discharged from Taihu Lake. The Taipu River plays a critical ecological role as an artificial river to increase the hydrological connectivity of the Taihu Lake basin [21]. Until the 1990s, the Taipu River had been the source for dredging and drainage in Taihu Lake. Later, it turned into one of the most crucial water supply rivers, with several water intake points located downstream [22]. With the change of river function from a drainage river to a water supply river, huge loads of heavy metals caused by previous industrial activities along the river have become the focus of attention. Previous studies on the Taipu river have suggested that the textile industry has caused heavy metal contamination, such as Sb contamination, in the water, sediment, and surrounding river soil, and has been considered the leading cause of pollution sources in downstream drinking water [23,24,25].

The Taipu River passes through three provinces of Jiangsu, Zhejiang, and Shanghai in China, running along with typical structures of agricultural, industrial, and residential areas. This river is considered a key source for transporting heavy metals in the river network owing to its dense network and abundant water resources. In addition, the Taipu River was classified as a Clear Water Corridor in 2018, thus, its water quality became a focus of attention for the three provinces. Further understanding of pollution sources is essential to maintain the water quality and function. With the transition of functions, there are several studies on heavy metals in the sediments of the Taipu River [24,25,26]. However, the spatial and temporal distribution of heavy metals derived from a single, small sampling scale are insignificant. What is more, the reason for spatial and temporal variability in heavy metals are relatively ambiguous when little attention is paid to hydrological conditions in such an environment. The main objective of this study is the analysis of heavy metals in the sediments of Taipu River and the interrelationship between metal concentrations and their influencing factors, making this study significant in providing scientific support for decision-makers.

## 2. Materials and Methods

### 2.1. Study Area

The Taipu River (30°59′–31°01′ N, 120°28′–121°04′ E) has the functions of hydrological scheduling, water supply, and navigation. One of the main hydrological operations is the opening of the Taipu Gate to release water from Taihu Lake through the Taipu River. With a length of 57.2 km, Taipu River is the largest artificial river in the Taihu Lake Basin. There are 40.5 km in Jiangsu, 1.46 km in Zhejiang, and 15.24 km in Shanghai. Thus, Jiangsu is the main city where the Taipu River is located. The bottom of the Taipu River is 128–150 m wide and has a water depth of 5.2–8.0 m. Taipu River is an important river link in the area, with an average annual flow of about 270 m^3^·s^−1^. There are many rivers and lakes connected to the Taipu River, such as Beijing-Hangzhou Canal (BHC), Cangzhou Dang (N1), Xueluo Yang (S1), Cao Dang (S2), Yingdou Lake (S3), and Yangjia Dang (S4). These lakes are surrounded by polders and hydrological conditions depend on the state of the gates. Furthermore, there are three drinking water intakes in the downstream of Taipu River, with a water supply scale of 351 × 10^4^ m^3^·d^−1^ [22].

### 2.2. Sample Collection and Analytical Methods

#### 2.2.1. Sample Collection and Pretreatment

**Figure 1:**
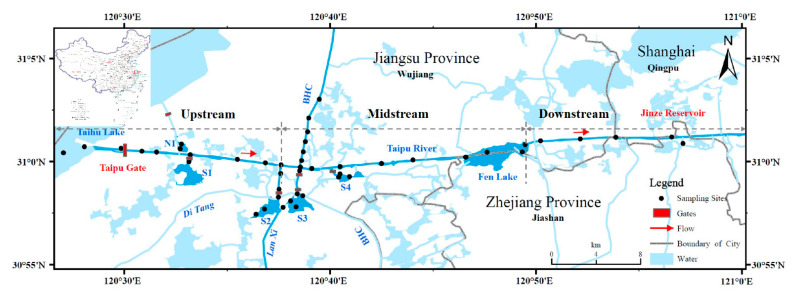
Location and sampling site.

The Taipu River experiences a distinct flood season and a non-flood season, and the flood season is from May to September each year. A total of 143 surface sediments were collected from Taipu River and its associated lakes in 2018, 2020, and 2021 during flood and non-flood seasons. Many industries along the river, including textile, printing and dyeing, machinery, and hardware, are potential contributors of heavy metals which become enriched in sediments [27]. Surface sediments were collected by a grab sampler based on the industrial distribution and the relationship between the river and lake, and handheld GPS was used to record the location (Figure 1). To prevent contamination, 3–5 cm of surface sediments and impurities were removed using a ceramic spoon. Finally, sediments were immediately placed into polyethylene bags and transported to the laboratory. In the laboratory, samples were frozen below −20 ℃ for 3–5 days, and dried with a freeze dryer. The dried samples were ground in an agate mortar, and sieved with a 200-mesh nylon sieve. Finally, samples were used the quartering method to take 10–20 g and store them at −20 ℃ for the chemical analysis.

#### 2.2.2. Analytical Methods

The concentration of heavy metals was determined by an aliquot (0.2 g) of grind and sieved sample digested by HNO_3_-HCl-HF-HClO_4_ in the Teflon tube. After cooling to room temperature, the digestion solution was diluted to 50 ml by 2% HNO_3_ and filtered with a 0.22 μm aqueous polyethersulfone filter membrane [28]. The concentrations of Sb, As, Cd, Cu, Pb, Cr, and Zn were determined by Inductively Coupled Plasma-Mass Spectrometry (ICP-MS: Agilent 7700, Agilent, Santa Clara, CA, USA). The method detection limits (MDLs) of metals are shown in Table S1. The Pb isotope ratio in digestions was detected by ICP-MS. The Pb isotope ratios of the Lead Isotopic standard (SRM 981, NIST, Gaithersburg, MA, USA) in measurements (^206^Pb/^207^Pb = 1.0933, ^208^Pb/^206^Pb = 2.1681) were very close to the standard value (^206^Pb/^207^Pb = 1.0934, ^208^Pb/^206^Pb = 2.1681).

The quality assurance and quality control (QA/QC) consisted of repeated analysis of reagent blanks, replicates, and spike recovery experiments representing 10% of total samples. To avoid contamination during the experiment, the acids used were Guaranteed Reagent. The containers used in the experiment were immersed in 10% HNO_3_ for at least 24 h, rinsed with Milli-Q water three times and dried. Precision and accuracy were verified using standard reference materials from the National Research Center for Geoanalysis of China (GBW07409). The results showed that the recovery rate ranged from 74.4% to 120% and the relative standard deviation (RSD) of three times repeated samples was within 15%, which satisfied the standard.

### 2.3. Geo-Accumulation Index

The geo-accumulation index (*I_geo_*) can compare the measured concentration of heavy metals with environmental background values, which can assess the degree of heavy metals contamination in sediments. The *I_geo_* value was calculated according to Equation (1):

(1) $$I_{geo}=\log_{2}\frac{C_{n}}{k\cdot B_{n}}$$

where *C_n_* is the measured concentration of heavy metal (mg·kg^−1^); *B_n_* represents the geochemical background of relevant metal (mg·kg^−1^). The Taipu River is located in the Taihu Lake Plain area and connects to Taihu lake. This study selected the common values in the plain of Taihu Lake, Jiangsu, China [29] as background values; *k* is the factor used to reflect the variation of rock formation, which generally is 1.5. The evaluation criteria for *I_geo_* are given in Table S2.

### 2.4. Source Identification and Apportionments

#### 2.4.1. Correlation Analysis

Correlation analysis is mainly used to analyze the dependence relationship between each heavy metal and discuss the degree of correlation. The correlation between different metals is generally determined by Pearson’s correlation coefficient and Spearman’s correlation coefficient. The larger the correlation coefficient, the greater the correlation between heavy metals, which means that they are likely to have the same source [30].

#### 2.4.2. Positive Matrix Factorization Model

The positive matrix factorization model (PMF) is a source analysis method developed by the U.S. Environmental Protection Agency (EPA) in the 1990s. PMF is more accurate than principal component analysis (PCA) for heavy metals source analysis, therefore PMF is commonly used for source analysis in sediment, soil, and atmosphere [19]. In the model, the sample data matrix (*X*) includes factor contribution matrix (*G*) and factor component matrix (*F*). The PMF model was defined as Equation (2) [31]:

(2) $$X_{ij}=\overset{p}{\underset{k=1}{\sum }}G_{ik}F_{kj}+E_{ij}$$

where *p* means the number of factors; *X_ij_* is the concentration of the *j*th metal in the *i*th sample; *G_ik_* is the contribution of the *k*th factor to the *i*th sample; *F_kj_* is the concentration of the *j*th metal in the kth factor; *E_ij_* is the residual of the *j*th metal in the *i*th sample. To obtain the optimal *G* and *F*, it is necessary to use the minimization objective function *Q*, which can be calculated by Equation (3):

(3) $$Q=\overset{n}{\underset{i=1}{\sum }}\overset{m}{\underset{j=1}{\sum }}\frac{E_{ij}^{2}}{U_{ij}^{2}}$$

where *n* and *m* denote the number of samples and metals; *U_ij_* is the uncertainty of the *j*th metal in the *i*th sample. The uncertainty is determined by the concentration of samples and method detection limit (MDL), which was expressed as Equation (4) [31]:

(4) $$U_{ij}=\{\begin{array}{c} \frac{5}{6}\times MDL\;\;\;\;\;\;\;\;\;\;\;\;\;\;\;\;c<MDL\\ \sqrt{{\left(\delta \times C\right)}^{2}+{\left(MDL\right)}^{2}}\;\;\;\;\;\;\;\;\;\;\;\;c\geq MDL \end{array}$$

where *C* is concentration of heavy metal; *δ* is relative standard deviation.

### 2.5. Data Analysis

Pearson correlation analysis, descriptive statistical analysis, and significant difference analysis were conducted by IBM SPSS 26 (IBM, Armonk, NY, USA). The sample data calculation, graphing, and heatmap were completed by Microsoft Excel 2016 (Microsoft, Redmond, WA, USA) and Origin 2018 (Origin Lab, Northampton, MA, USA). The map of the sampling site was produced by ArcGIS 10.2 (ESRI, Redlands, CA, USA). Source apportionment of heavy metals was obtained from PMF 5.0 (EPA, Washington, DC, USA).

## 3. Results

### 3.1. Heavy Metal Concentrations in Sediment

Table 1 summarizes heavy metal concentrations in surface sediments collected from Taipu River, all of which exceeded MDL. The results indicated that the sediments had the highest concentration of Zn, with a mean of 154.75 mg·kg^–1^, followed by Cu and Cr. It was found that except for Cr and As, the mean concentrations of the remaining metals were higher than the background values in the plain of Taihu Lake, Jiangsu, China [29]. The exceedance rates of metals were as follow: Cd > Pb > Cu > Sb > Zn > As > Cr. Among all metals, the exceedance rates of Cd, Pb, Cu, Sb, and Zn were above 90%. Additionally, the I_geo_ value obtained for Cd (−1.06–2.44), Sb (−1.39–2.20), Cu (0.32–3.63), Zn (−1.86–1.86), and Pb (0.27–1.03) indicated that these metals were at light-medium pollution (Table S3).

Table 2 shows the heavy metal concentrations in river sediments around the world over the last 20 years. Comparatively, the concentrations of heavy metals in the present study showed a decreasing trend, in particular, in the concentration of Sb. The Taipu River is the river that connects Taihu Lake to the Huangpu River. The concentrations of Sb, Cd, As, and Cr in this study were lower than those in Taihu Lake [3,32] and Huangpu River [33], while Cu, Zn, and Pb were generally higher than in these rivers. In particular, the concentrations of Sb, Cd, Cr and Cu were lower than the reported values in the previous study by Wang et al. [24], in which five sediment samples were collected in 2015 and the point locations were overlapped in this study. Moreover, the concentrations of heavy metals in the Taipu River were higher than those in the Yellow River [34], while significantly lower (*p* < 0.05) than those in the sediments of the Pearl River [35], the Xiang River [30], the St. Lawrence River in Canada [36], and the Danube River in Germany [37], all of which are surrounded by industry and agriculture. Overall, the Taipu River sediments contain low concentrations of heavy metals. However, as the upstream of the drinking water source, the control of heavy metals in the Taipu River should be more stringent.

### 3.2. Heavy Metal Distribution Characteristics

#### 3.2.1. Temporal Distribution of Heavy Metals

**Figure 2:**
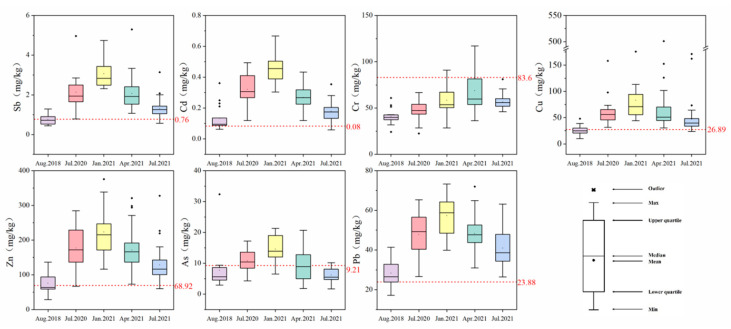
Temporal distribution of heavy metals in sediments (The red dashed line represented the background value of each heavy metal).

Heavy metals in sediments collected in September 2018 and July 2021 showed significant differences from those collected in July 2020, January 2021, and April 2021 (*p* < 0.05). According to the hydrological situation of Taihu Lake, the flood season of the Taihu Lake basin is from May to September every year, and the non-flood period is the rest of the year. The heavy metal concentrations for each season were analyzed, and it was concluded that the order of metals concentration was non-flood season > flood season (Figure 2). This order was consistent with previous reports in Taipu River [24]. Supportively, Chen et al. [15] found higher concentrations of heavy metals in the sediments of the Three Gorges Dam in winter than in summer. However, only some metals were found to have seasonal variation in the Le’an River in China, while no significant seasonal variation was found in the Lhasa River in Tibet [43,44]. Seasonal variations in heavy metal concentrations are not prevalent, so it was necessary to further analyze the causes.

As can be observed from Figure S1, there were some differences in the temporal distribution characteristics of heavy metals in the mainstem (MS) and connected lakes (CL). The MS showed significantly higher concentrations of all metals in the non-flood season (e.g., January and April) than in the flood season (e.g., July), except Cr. In contrast, the CL (e.g., S2 and S3) showed no significant variation in the heavy metal concentrations between the flood and non-flood seasons.

The river network in the Taihu Lake basin is a complex "natural–artificial" water system, and the hydrological condition of the river is based on the water conservancy project schedule [21]. The Taipu River plays a significant role in the dredging and drainage of Taihu Lake and is responsible for 40% of the drainage of the Taihu Lake basin each year. Therefore, the hydrological condition should also be considered in the sediments of the Taipu River to obtain reliable outcomes. Considering the drainage and flow rate of the Taipu Gate and the precipitation in the Lake Tai basin (Figure S2), the average flow rate was 701.7 m^3^·s^–1^ and 104.0 m^3^·s^–1^ in July 2020 and July 2021, respectively [45]. On the contrary, in September 2018, January 2021, and April 2021, the rates were only 79.2 m^3^·s^–1^, 60.2 m^3^·s^–1^, and 81.5 m^3^·s^–1^, respectively. It was obvious that the maximum and minimum flow rates occurred during the flood season and non-flood season, respectively. The drainage and precipitation during the flood season were also found to be remarkably higher than that in the non-flood season. In addition, it has been shown that rainfall events affect particulate matter export, with lower precipitation producing higher particulate matter loads [46]. The lowest flow rates and precipitation in MS (Figure S2) were recorded in January 2021 when heavy metal concentrations were at their highest (Figure S1), suggesting that low flow rates may facilitate the deposition of heavy metals with suspended particles [15].

Hydrological conditions promoted sediment resuspension, and the heavy metal concentrations in suspended sediment showed a significant positive correlation with the amount present in the sediment [47]. According to previous studies, the concentration of suspended particles of the Taipu River was substantially higher during the non-flood season (53.79 mg·L^–1^) than that during the flood season (32.79 mg·L^–1^) [24].The drainage of Taipu Gate during the flood season (14.74 × 10^8^ m^3^) is much larger than that of the non-flood season (1.63 × 10^8^ m^3^). When the Taipu Gate was opened during the non-flood season, quantities of fine-grained sediments stored in the upstream Taihu Lake were released into the Taipu River, resulting in the finer grain size of its surface sediments [48]. The low flow rate and high suspended particle concentration were conducive to the deposition of heavy metals, resulting in the enrichment of heavy metals in the sediment during the non-flood season [15]. In addition, sediment redox conditions can also affect the accumulation of heavy metals, which change with river flow rate. As the flowing river was a paramount medium for transporting heavy metals, it was tentatively assumed that changes in river flow velocity, due to the opening of Taipu Gate, would have an impact on the distribution of heavy metals in the sediments.

#### 3.2.2. Spatial Distribution of Heavy Metals

Including MS, the rank of total heavy metal concentrations was in the following order S3 > S2 > S4 > MS > N1 > BHC > S1 (Table S4 and Figure S3). It was found that heavy metal concentrations in the sediments of the Taipu River were roughly lower than in Taihu Lake (Table 2). A large amount of sediment was trapped during the closure of the Taiping Gate [21], which led to the conclusion that the impact of heavy metals in Taihu Lake on the Taipu River was negligible. The spatial distribution of Sb, Cd, Cu, Zn, and Pb in the sediment is shown in Figure S4. The concentrations of heavy metals in midstream were highest compared to the other parts of the river, where the average values of Sb, Cd, Cr, Cu, Zn, As, and Pb were 1.85 mg·kg^−1^, 0.29 mg·kg^−1^, 53.15 mg·kg^−1^, 72.09 mg·kg^−1^, 165.83 mg·kg^−1^, 8.61 mg·kg^−1^, and 44.64 mg·kg^−1^, respectively. The midstream is densely distributed with industries, while those around downstream are scattered. On the other hand, the midstream is connected to the BHC and CL with high concentrations of heavy metals. Based on the above, it was concluded that the heavy metals in the Taipu River mainly originated from the midstream. The higher concentrations of heavy metals in the midstream could be attributed to the multiple influences of anthropogenic emissions and the confluence of rivers.

The Taipu River and lakes have a relatively gentle topography with small breakage areas, and the river runoff is reciprocal [21]. Previous research has demonstrated that adequate hydrologic connectivity promotes metals mobility in sediment [49], and thus the heavy metals coefficients of variation in rivers are often larger than those in lakes [50]. However, S2 and S3 had a high coefficient of variation and a significant difference (*p* < 0.05) from MS, implying that there might be other sources of heavy metals in S2 and S3.

**Figure 3:**
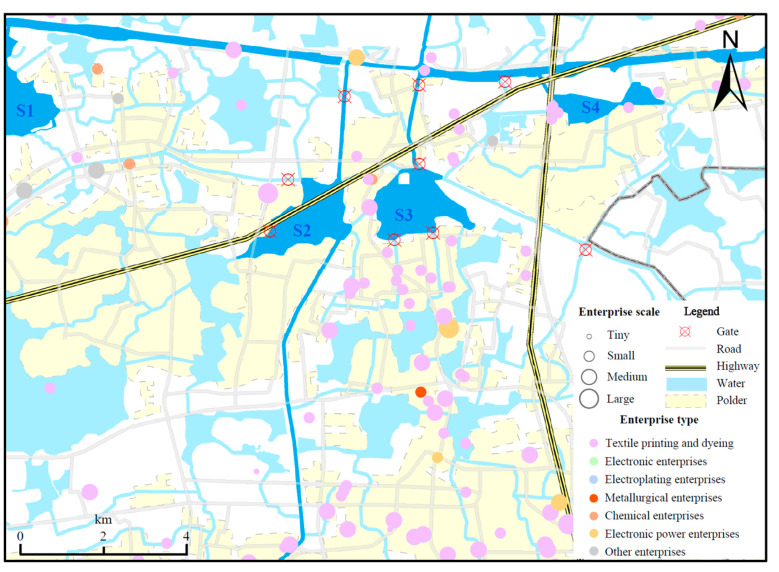
Spatial distribution of industrial enterprises, transport, and gates around the S2, S3.

Based on Figure S4 and Table S4, the average concentrations of Sb and As were 0.98 mg·kg^−1^, 2.98 mg·kg^−1^ in S1, 2.10 mg·kg^−1^, 11.54 mg·kg^−1^ in S2, and 2.46 mg·kg^−1^, 13.28 mg·kg^−1^ in S3. In summary, the concentrations of heavy metals in S2 and S3 were 2–5 times higher than in S1, suggesting that S2 and S3 were predominant lakes for heavy metals enrichment in that region. According to a survey, S2 and S3 are important catchments for the river water from Di Tang, Lan Xi, and BHC. Moreover, the three rivers are located in the towns of Pingwang and Shengze, which are important industrial production centers in the Wujiang District (Figure 3). These heavy metals (e.g., Sb, Cd, Cu, Zn, and Pb), as the characteristic pollutants from nearby industries [40,51,52], were transported with the river, and S2 and S3 have become transit points for heavy metals accumulation.

**Figure 4:**
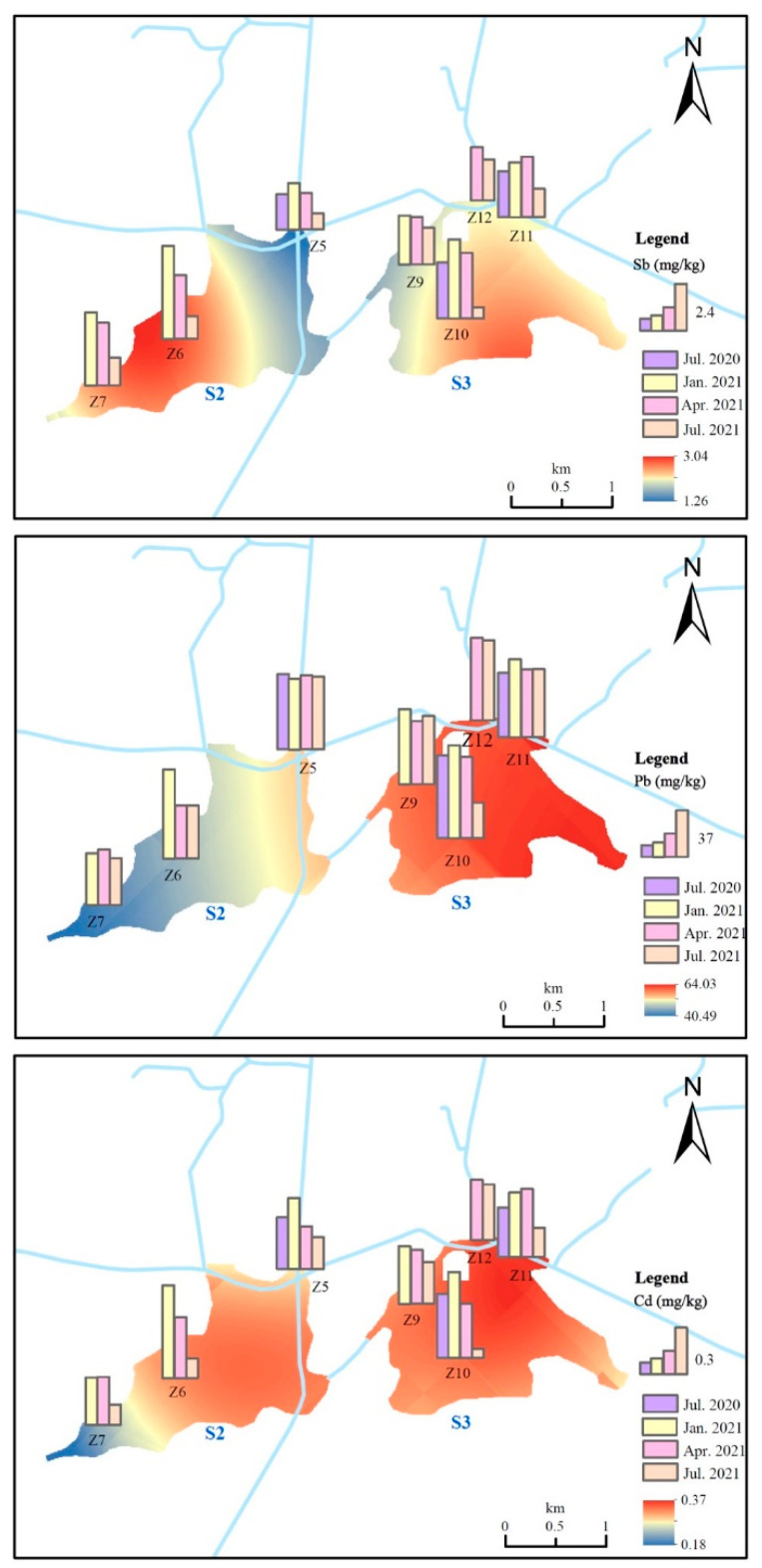
Spatial and temporal distribution of Sb, Cd, and Pb in S2, S3 sediments.

Wujiang District is a typical polder terrain, and the location of the polder gates is a critical factor influencing the spatial distribution of metals [53]. The polder gates play a significant role in preserving the water quality within the polder, with S2 and S3 being the preferred water discharge site from the rivers. It can be observed from Figure 3 and Figure 4 that the sites with high heavy metal concentrations in these two lakes were near specific gates, such as Z6 and Z10. In addition, the distribution of industrial enterprises and transportation networks around S2 and S3 (Figure 3) revealed that the enrichment of heavy metals in sediments was caused by a large number of textile industries and electronic power industries [54,55]. Furthermore, a high concentration of Pb was observed at the Z10 and Z11 sites, complying with previous reports [56]. Site Z10 was near to a complex traffic network (Figure 3) and Z11 was on the BHC. The BHC is an essential transportation channel, with an annual freight volume of over 500 million tons, ranking fourth in the world. It was confirmed that during the shipping process, Pb was continuously discharged into the river, resulting in substantial Pb enrichment in the sediments [32].

### 3.3. Source Apportionment of Heavy Metals

To obtain a credible and accurate interpretation of heavy metal sources, correlation analysis and the PMF model were employed, based on which the Pb isotope ratio can indicate the source of Pb more accurately. The result of Pearson correlation analysis showed that there were significant correlations among each metal (*p* < 0.01). As can be observed from Figure S5, Pb-Zn (r = 0.83), Cd-Zn (r = 0.79), and Sb-Cd (r = 0.76) presented a positive correlation, suggesting that the origin of these pairs of metals could be from the same sources.

**Figure 5:**
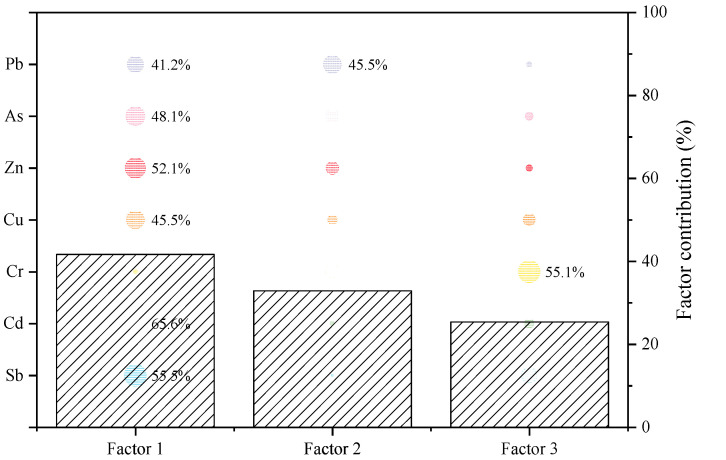
Classification results of PMF model (Different color-sized circles were used to indicate the proportion of each heavy metal in the different factors).

The concentrations in the present study were introduced into the PMF model and the model was run 20 times with 3–5 factors to determine the number of factors with the minimum Q (Equation (3)). Based on the results of the processing PMF, three factors were identified. Factor 1 had the highest contribution and was mainly dominated by Cd (65.6%), Sb (55.5%), Zn (52.1%), As (48.1%), Cu (45.5%), and Pb (41.2%). Factor 2 had a strong relation with Pb (45.5%). Factor 3 was dominated by Cr (55.1%) (Figure 5). In general, an element with equivalent loadings in multiple components was considered to have multiple sources, such as Pb in this study.

Heavy metals, such as Cd, Sb, Zn, As, Cu, and Pb, were concentrated in Factor1. Among all, Sb is a characteristic heavy metal in the dyeing and textile industry [57]. As per the literature, antimony-containing compounds, such as Sb_2_O_3_, Sb(CH_3_OO)_3_, and Sb_2_(OCH_2_CH_2_O)_3_, are essential condensation catalysts in the polyester fiber production process [58]. These compounds have poor solubility in water and tend to retain in polyester fibers, and a strong alkali treatment washes away Sb left in the polyester fibers, releasing Sb into the wastewater. Besides, Sb is also contained in dyes and auxiliaries in dyeing and printing processes [58]. Eventually, Sb is discharged into the river as Sb^3+^ and Sb^5+^. According to the previous studies, the total emission of Sb in Taipu River was 5877.41 kg/a, and Sb was enriched in the sediment by adsorption of metal oxides [59,60]. The electroplating industry has mainly been reported to release heavy metals, such as Cd, Zn, Co, and Ni [5]. At the same time, Cd is widely used as a raw material for industrial color mixing pigments, metal manufacturing, and rubber manufacturing [18]. According to the 2019 yearbook data, Wujiang District had 577 textile industrial enterprises and 148 computer manufacturing industrial enterprises with a gross product of 45.1 billion and 71 billion yuan, respectively. Excessive industrial activities have led to the enrichment of heavy metals in sediments. Therefore, it was inferred that Factor1 was mainly influenced by industries such as the printing and dyeing textile industry, electroplating, and machinery manufacturing.

**Figure 6:**
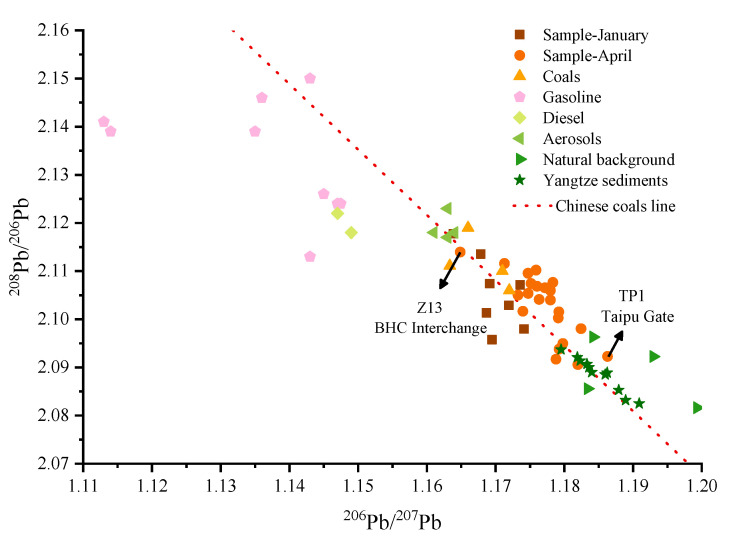
Lead isotope ratios (^206^Pb/^207^Pb vs ^208^Pb/^206^Pb) in sediments and citations (data from Table S3). The data of regression line for Chinese coals was from Bi et al. [64].

Higher loadings of Pb were found in Factor 2. Moreover, the Pb had a relatively low coefficient of variation, suggesting emissions from surface or mobile sources might be present. Since Pb is commonly used in tires, fences, lubricants, and other parts, it is often considered to be a characteristic constituent of traffic pollution [61]. This result was combined with the Pb isotope ratio analysis to elucidate the potential source of Pb. The isotopic ratios of Pb in Taipu River sediments ranged from 1.163 to 1.186 for ^206^Pb/^207^Pb and from 2.091 to 2.118 for ^208^Pb/^206^Pb (Figure 6, Table S5). The ^206^Pb/^207^Pb ratio of sediments in the Taipu gate site closest to Taihu Lake was consistent with the natural background value ratio, from [62]. In contrast, the ^206^Pb/^207^Pb ratios were different from the natural background in other sites (^206^Pb/^207^Pb: 1.183–1.199, ^208^Pb/^206^Pb: 2.082–2.096) and Yangtze River sediments (^206^Pb/^207^Pb: 1.180–1.190, ^208^Pb/^206^Pb: 2.083–2.094) [62,63], suggesting that the Pb in sediments might have originated from anthropogenic activities. The literature survey indicates that the Pb isotope ratios in sediments were between those of gasoline, diesel, coal, and natural sources from Jiangsu and Shanghai [64,65]. Moreover, the Pb isotope ratio at the BHC interchange (Z13) with heavy traffic and shipping was almost close to those of gasoline and diesel (^206^Pb/^207^Pb: 1.130–1.149, ^208^Pb/^206^Pb: 2.113–2.151). The Pb isotope ratio of Z13 and the spatial distribution of Pb revealed that the BHC interchange sample was indicative of particulate matter originating from traffic [32,66]. The increasing traffic has led to the gradual settling of the exhaust gas containing Pb, which has become enriched in the sediment. Therefore, it was presumed that Factor 2 was particle deposition caused by traffic.

Factor 3 corresponded to high loading values of Cr (55.1%). Correlation analysis showed that Cr had no significant correlation with the rest of the metals. The coefficient of variation for Cr in the sediments was low and showed a normal distribution in the Taipu River, indicating that the spatial and temporal distribution of Cr was relatively uniform. Furthermore, the overall concentration level of Cr was lower than the background value. The Taipu River is located in a yellow-brown loam soil zone containing high concentrations of Cr and Mn compared to Chinese background values [29]. This suggested that Factor 3 may be attributed to sediment parent composition and is consistent with previous reports [26].

## 4. Discussion

### 4.1. Influence of Hydrological Conditions on Heavy Metals

In the present study, it was found that the synergistic effects of flow rate changes caused by gate operation and precipitation influence the spatial and temporal distribution of heavy metals. The hydrological conditions are crucial in the enrichment of heavy metals in Taipu river sediments [11]. In turn, hydrological conditions are affected by precipitation and gate status. A significant and robust positive correlation between the drainage from the Taipu Gate, the precipitation in the Taihu Lake basin, and the Taipu River flow rate are evidenced in Figure S5. In contrast, all the above hydrological factors negatively correlated with the heavy metal concentrations in the sediments.

The mobility of heavy metals and their behavior in sediments is affected by the hydrological and depositional conditions of the river [12]. With the influence of hydrodynamic conditions, both the physical properties and the chemical environment of the sediment are changed, which, in turn, influences heavy metal accumulation. The particle size distribution of the sediment is closely related to hydrological conditions, and various particle sizes show different sorption capacities for heavy metals [11]. In general, fine particles adsorb more heavy metals than coarse particles, influencing the concentration of heavy metals in the sediments [11,12,67]. Further, the bottom sediments can resuspend in water by strong hydrodynamics, and fine particles remain in the water and are carried away by the current, along with heavy metals. It has been found that resuspension can reduce heavy metal concentrations in sediments by 24% [36]. The Taipu River sediments were reported to be predominantly slit, which may correlate with river scour [26].

Although hydrological conditions have a significant influence on the distribution of heavy metals, the mobility of heavy metals is also influenced by redox conditions. Noteworthy is the fact that the redox conditions in the sediment are altered by the hydrological conditions, affecting the transformation and release of heavy metals in the sediment [11]. The surface sediment in the river is a chemical and biological system that is extremely sensitive to redox conditions [68]. The studies have reported that metals, such as Cu, Zn, and Cd, are more likely to migrate in oxygenated environments [69]. During the flood season, the Taipu Gate was opened to divert water to control the water level in the Taihu Lake basin, significantly increasing the flow and promoting an oxygenated environment. Furthermore, Sb was stably present in the sediment in the residual state, and its concentration in the non-flood season was higher than in the flood season in Taipu River [59]. During the non-flood season, the sediments were less disturbed due to weak hydrological conditions. The sediment redox conditions were stable under low flow rates, and Sb release capacity was weakened, contributing to the stable enrichment of Sb in the sediment in the form of residue. On the other hand, a high-flow environment during the flood season promotes an oxygenated environment in the sediments, which leads to the oxidation of Sb^3+^ to Sb^5+^. These Sb^5+^ salts are easily soluble in water and migrate with water in the dissolved state, attenuating enrichment in the sediments [70]. The mobility of heavy metals in aquatic environments is complex. The redox conditions of sediments play an important role in the process of heavy metal mobility, and their interrelationship deserves further study.

### 4.2. Influence of Industrial Activities on Heavy Metals

Although strict industrial controls have been implemented, industrial distribution can still be considered a non-negligible source of heavy metals. It was shown in Figure S6a that the industrial development of Wujiang began with the silk industry and rapidly expanded in three decades. Hence, textile industries in Wujiang are worth noting, due to their large number, and they account for about 34.3% of industrial capital in Wujiang (Figure S6b). Since the raw materials and catalysts in the textile industry are rich in heavy metals, their concentration also gradually increased in the sediments during the developing industrial activities. There were many reports about incidents wherein Sb concentration anomaly frequently occurred in Taipu River during 2014–2018 [60]. The concentration of Sb in N1 was as high as 5.29 mg·kg^–1^, which is much higher than that in Jiangsu soil (0.77 mg·kg^–1^) and Taihu Lake sediment (2.37 mg·kg^–1^) [29,32]. To improve the water quality, Wujiang District carried out the rectification of the textile industry in 2018. Based on the Sb concentrations measured in 2015, 2020, and 2021, a progressive downward trend in Sb concentrations in the sediments was observed. However, Sb was still accumulating in the environment as the discharge concentration of Sb in the wastewater (0.1 mg L^–1^) [71] was still higher than that in the river.

In addition to Sb, Pb showed significant temporal differences in the MS of the Taipu River, while the differences were not predominant in S2 and S3, suggesting that hydrological conditions were not the only factor influencing the distribution of Pb. In particular, the concentration of Pb was significantly higher in S3 and evenly distributed throughout the seasons, which was likely caused by the busy shipping on the BHC. Considering the regional characteristics and source analysis, it was presumed that the dense road traffic around S3 was one of the factors contributing to the deposition and accumulation of Pb in the sediment. In addition, with the increase of impermeability, due to industrialization, heavy metals from traffic sources are more likely to enter the rivers with surface runoff [72].

Moreover, there are a number of polders distributed around the Taipu River, and the rivers within the polders receive tail water from industrial wastewater treatment plants. Based on Figure 3, the distribution of heavy metals in S2 and S3 near the polder gates revealed high concentrations of several heavy metals at these sites, implying that the water quality within the polder affects the heavy metal content in CL. Therefore, it can be concluded that the industrial emissions within the polder are the fundamental factors contributing to heavy metal enrichment in the sediments. 

## 5. Conclusions

In this study, the influence of hydrological conditions and industries on the distribution of heavy metals was examined according to sediment samples from the Taipu River in 2018, 2020 and 2021. The spatial and temporal distribution of heavy metals was heterogeneous. The significantly higher concentrations were in the midstream (including S2 and S3) during the non-flood season (*p* < 0.05), with Sb at 2.46 mg·kg^−1^ and Pb at 56.9 mg·kg^−1^. It was also clear that the drainage process of Taipu Gate and precipitation had a profound impact on the hydrological conditions and, thereby, the accumulation of heavy metals in rivers. By combining the PMF model and industrial distribution patterns, Sb discharged through textile wastewater was still transported in a manner dependent on hydrological conditions and enriched in the environment. It could be concluded that the main anthropogenic factor affecting heavy metals spatial distribution were industrial activities and traffic activities within the polder. 

The present study clarified the influence of industrial and traffic sources on the spatial and temporal distribution of heavy metals under complex hydrological conditions in a plain water network area. The data from the study can provide basic river data and help to formulate environmental management policies. It is also proposed that hydrological conditions should be taken into account in the management of heavy metals.

## Figures and Tables

**Table 1:** Heavy metals concentration (mg·kg^−1^) in sediments (*n* = 143).

	Sb	Cd	Cr	Cu	Zn	As	Pb
Max	5.29	0.67	117.14	501.08	375.42	32.35	73.23
Min	0.44	0.06	22.58	9.83	28.52	1.72	17.18
Mean	1.83	0.26	57.36	61.12	154.75	8.97	44.86
Median	1.65	0.24	55.06	47.73	142.21	8.19	44.86
SD	0.94	0.13	17.15	54.76	67.81	5.00	12.08
VC (%)	51.38	48.75	29.89	89.60	43.82	55.73	26.93
GB	0.77	0.08	83	27	69	9.2	23.9
ER (%)	91.6	98.6	8.4	92.3	91.6	37.1	96.5

Abbreviations: SD, standard deviation; VC, variation coefficient, GB, the geochemical background of heavy metals in the plain of Taihu Lake, Jiangsu, China; ER, exceedance rate.

**Table 2:** The summary of heavy metals average concentration (mg·kg^−1^) in sediments.

	Sb	Cd	Cr	Cu	Zn	As	Pb	References
Taipu River, China, 2021	1.83	0.26	57.36	61.12	154.75	8.97	44.86	Present study
Taipu River, China, 2015	7.74	0.74	87.20	62.20	/	/	34.90	[24]
Taihu Lake, China, 2018	/	0.61	68.85	35.53	109.32	16.99	29.70	[3]
Taihu Lake, China, 2015	2.37	0.55	82.30	32.80	109.0	/	35.10	[32]
Huangpu River, China, 2018	2.80	2.20	96.30	40.20	139.7	11.30	68.60	[33]
The Yellow River, China, 2020	/	0.11	45.31	15.59	49.40	11.74	17.71	[34]
Three Gorges Reservoir, China, 2014	/	1.17	114.8	95.81	164.89	/	73.92	[38]
Jialu River, China, 2009	/	2.93	60.80	39.22	107.58	6.31	29.35	[39]
The Pearl River, China, 2011	/	3.77	180.6	182.5	487.12	/	150.61	[35]
Xiangjiang River, China, 2010	/	15.0	51.99	43.01	266.57		71.10	[30]
Lianjiang River, China, 2005	/	4.10	/	1070	324.0	/	230.0	[40]
Zijiang River, China, 2017	36.6	3.00	67.51	34.19	141.90	31.53	35.68	[41]
St. Lawrence River, Canada, 2016	/	0.80	68.50	108.0	3035.9	8.80	58.20	[36]
Nador lagoon, Morocco, 2013	/	1.60	71.60	10.20	554.9	/	135.0	[42]
Danube River, Germany, 2001	/	1.50	71.10	/	258.0	20.10	52.5	[37]

## Data Availability

The data used to support the findings of this study are available from the corresponding author upon request (lifeipeng@usst.edu.cn).

## Supplementary Materials

The following supporting information can be downloaded at: https://www.mdpi.com/article/10.3390/ijerph191610116/s1, Table S1. The MDL value (mg·kg^−1^) of heavy metals; Table S2. The I_geo_ value level classification criteria; Table S3. The I_geo_ value of sediments in Taipu River; Table S4. Heavy metals average concentration (mg·kg^−1^) in part of Taipu River; Table S5. Lead isotopic compositions in several material sources from China; Figure S1. Temporal distribution of heavy metals in sediments of the Taipu River MS, S2, S3; Figure S2. Variations of precipitation, drainage, and flow rates in the Taipu River; Figure S3. Spatial distribution of heavy metals in sediments; Figure S4. Spatial distribution of Sb, Cd, Cu, Zn, and Pb in Taipu River sediments; Figure S5. Pearson correlations among target metal elements and hydrological conditions in the Taipu; Figure S6. (a) Temporal trends of industrial and major industrial output values in Wujiang from 1990 to 2019 (b) Distribution of industrial enterprise types in Wujiang. Refs. [62,63,64,65,73,74] have been cited in the supplementary file.
