# Supplementary material for: Co-Effects of Hydrological Conditions and Industrial Activities on the Distribution of Heavy Metal Pollution in Taipu River, China
Source: Int J Environ Res Public Health. 2022 Aug 16;19(16):10116. doi: 10.3390/ijerph191610116 (PMC9407723; doi:10.3390/ijerph191610116)
Supplement: Supplementary file 1 [file ijerph-19-10116-s001.zip › ijerph-1854128-supplementary.pdf]

Supplementary Material

Co-effects of hydrological conditions and industrial activities on the distribution of heavy metal pollution in Taipu River, China

Qinglu Yao [1], Ling Chen [1,2] Lingchen Mao [3], Yu Ma [1], Fengyan Tian [3], Ruijie Wang [3], Xiangzhou Meng [1,4],and Feipeng Li [3,*]

1 College of Environmental Science and Engineering, Tongji University, Shanghai 200092, China
2 State Key Laboratory of Pollution Control and Resource Reuse, Tongji University, Shanghai 200092, China
3 School of Environment and Architecture, University of Shanghai for Science and Technology, Shanghai 200093, China
4 Shanghai Institute of Pollution Control and Ecological Security, Shanghai 200092, China
* Correspondence: lifeipeng@usst.edu.cn

Table S1. The MDL value (mg·kg^{-1}) of heavy metals.

	Sb	Cd	Cr	Cu	Zn	As	Pb
MDL	0.02	0.01	8.1	0.19	24	17	0.10

Table S2. The I_{geo} value level classification criteria.

Range	Degree
$I_{geo} \leq 0$	No pollution
$0 < I_{geo} \leq 1$	Light- Medium pollution
$1 < I_{geo} \leq 2$	Medium pollution

Table S3. The I_{geo} value of sediments in Taipu River.

	Sb	Cd	Cr	Cu	Zn	As	Pb
Mean	0.48	0.89	−1.18	0.32	0.44	−0.84	0.27
Max	2.20	2.44	−0.09	3.63	1.86	1.23	1.03
Min	−1.39	−1.06	−2.46	−2.04	−1.86	−3.00	−1.06

Table S4. Heavy metals average concentration ($mg \cdot kg^{-1}$) in part of Taipu River.

		Sb	Cd	Cr	Cu	Zn	As	Pb
MS	Upstream	1.58	0.23	50.78	48.12	113.29	7.36	38.50
	Midstream	1.85	0.29	53.15	72.09	165.83	8.61	44.64
	Downstream	1.34	0.19	56.19	51.14	135.07	6.14	40.98
CL	N1	3.35	0.16	57.71	40.86	121.80	8.71	36.99
	S1	0.98	0.15	64.69	41.11	101.72	2.98	30.33
	S2	2.10	0.29	63.46	48.01	179.78	11.54	51.27
	S3	2.46	0.34	72.29	54.89	231.55	13.28	56.90
	S4	1.99	0.28	63.77	157.83	232.90	10.21	53.41

Table S5. Lead isotopic compositions in several material sources from China.

Sample	$^{206}Pb/^{207}Pb$	$^{208}Pb/^{206}Pb$	References
Sample-April, 2021	1.169	2.101	Present study
	1.169	2.107	
	1.174	2.098	
	1.169	2.096	
	1.164	2.118	
	1.172	2.103	
	1.168	2.114	
	1.174	2.107	
Sample-January, 2021	1.176	2.110	Present study
	1.186	2.092	
	1.179	2.094	
	1.180	2.095	
	1.176	2.107	
	1.179	2.102	
	1.179	2.100	
	1.179	2.092	
	1.171	2.112	
	1.175	2.110	
	1.174	2.102	
	1.176	2.104	
	1.178	2.106	
	1.178	2.108	
	1.173	2.105	
	1.175	2.107	
	1.165	2.114	
	1.182	2.091	
	1.175	2.105	
	1.177	2.107	
	1.182	2.098	
	1.178	2.104	
Coals-Jiangsu	1.172	2.106	[1]
	1.171	2.110	
	1.166	2.119	
Coals-Shanghai	1.163	2.111	[1]
Gasoline-Shanghai	1.143	2.150	[2]
	1.135	2.139	
	1.136	2.146	[3]
	1.147	2.124	
	1.143	2.113	
	1.145	2.126	
	1.147	2.124	[1]
	1.114	2.139	
	1.113	2.141	
Diesel	1.149	2.118	[4]
	1.147	2.122	
	1.148	2.135	
	1.163	2.123	

Sample	$^{206}Pb/^{207}Pb$	$^{208}Pb/^{206}Pb$	References
natural background	1.183	2.086	[5]
	1.184	2.096	
	1.199	2.082	
	1.193	2.092	
Yangtze sediments	1.184	2.090	[6]
	1.180	2.093	
	1.184	2.089	
	1.186	2.089	
	1.188	2.085	
	1.189	2.083	
	1.182	2.092	
	1.191	2.083	
	1.182	2.091	
	1.186	2.089	
	1.183	2.091	

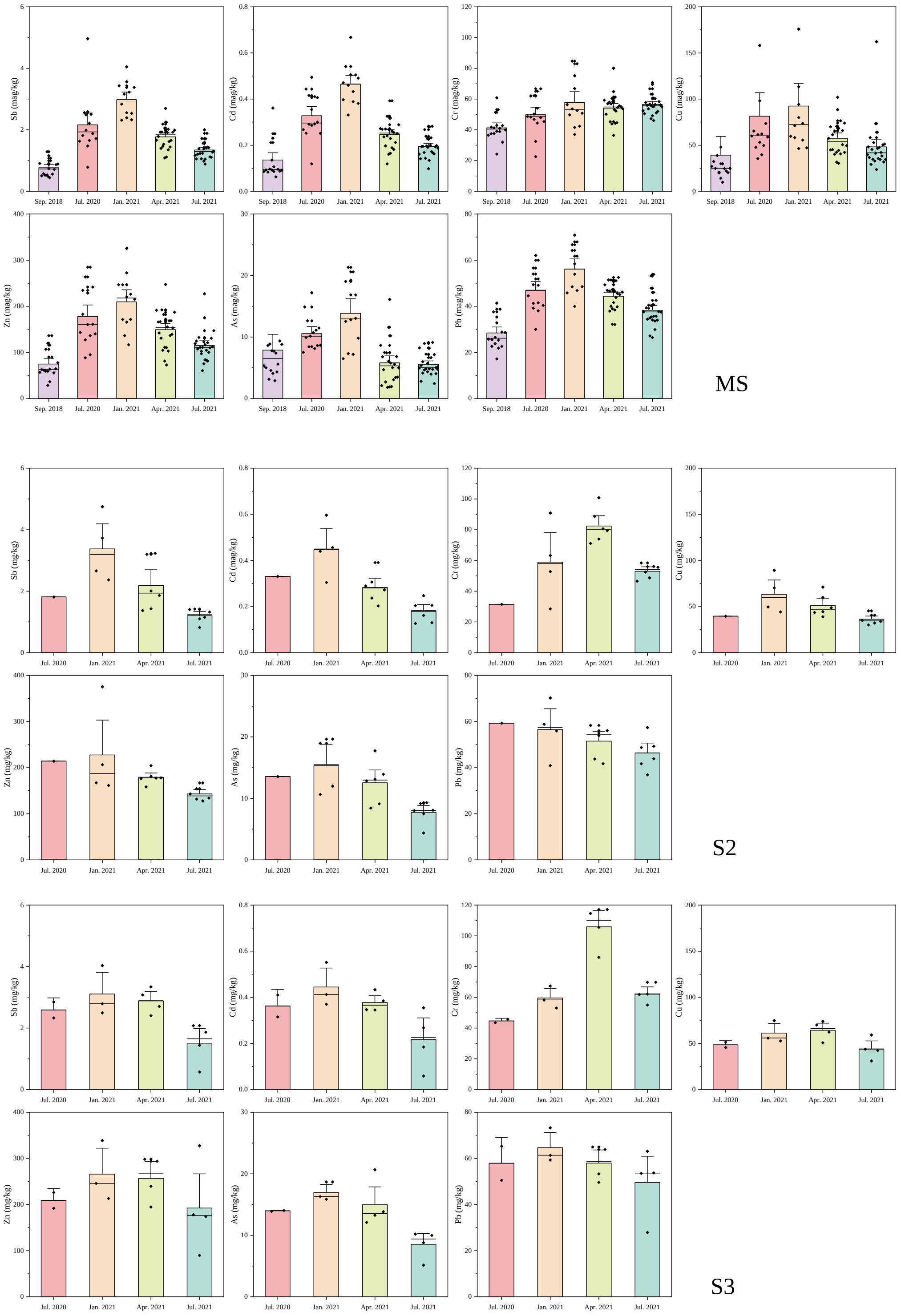

Figure S1. Temporal distribution of heavy metals in sediments of the Taipu River MS, S2, S3.

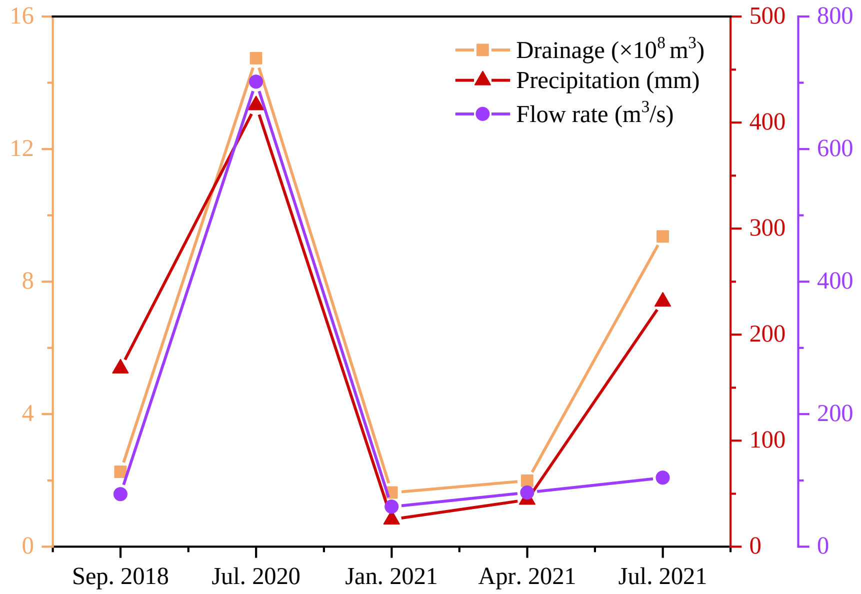

Figure S2. Variations of precipitation, drainarge, and flow rates in the Taipu River.

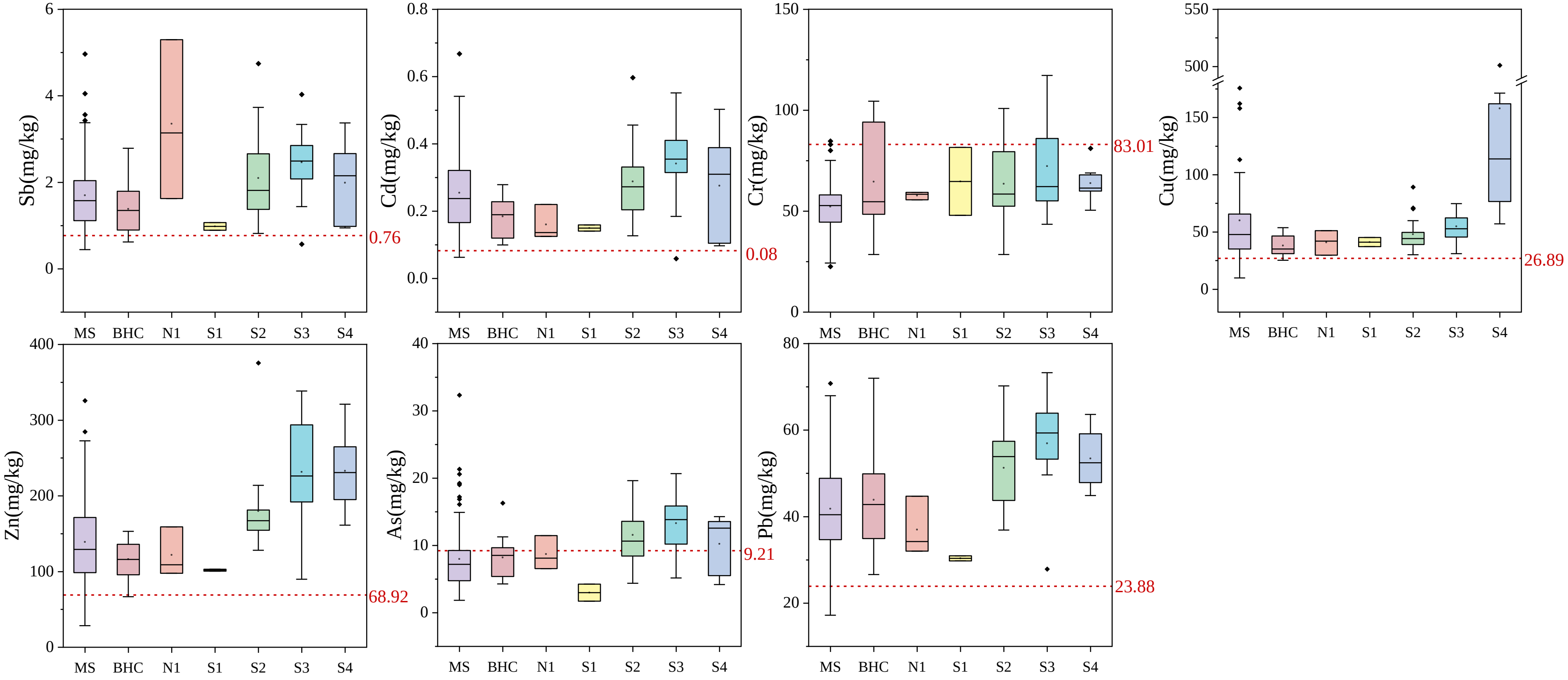

Figure S3. Spatial distribution of heavy metals in sediments (The red dashed line represented the background value of each heavy metal).

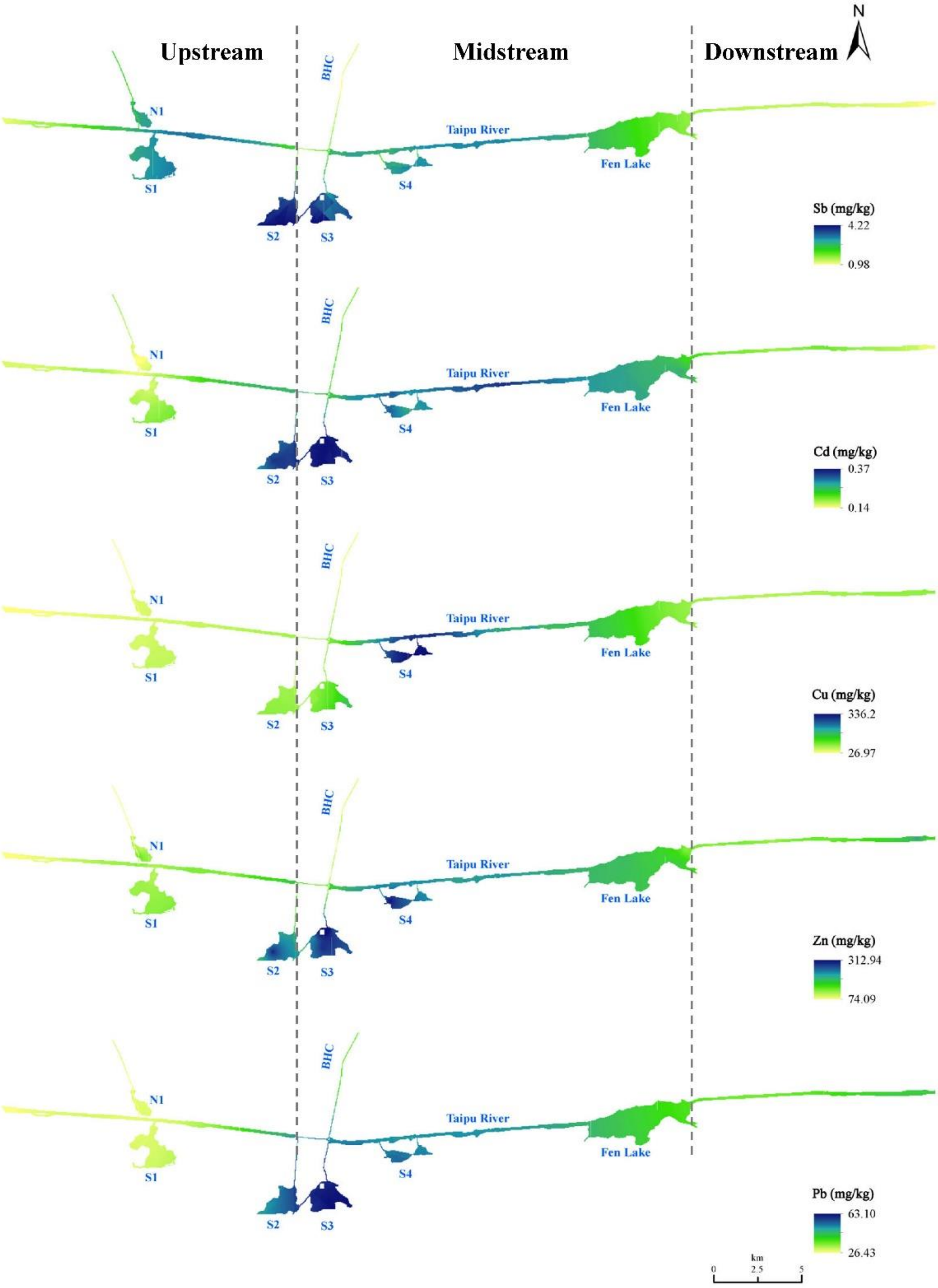

Figure S4. Spatial distribution of Sb, Cd, Cu, Zn, and Pb in Taipu River sediments.

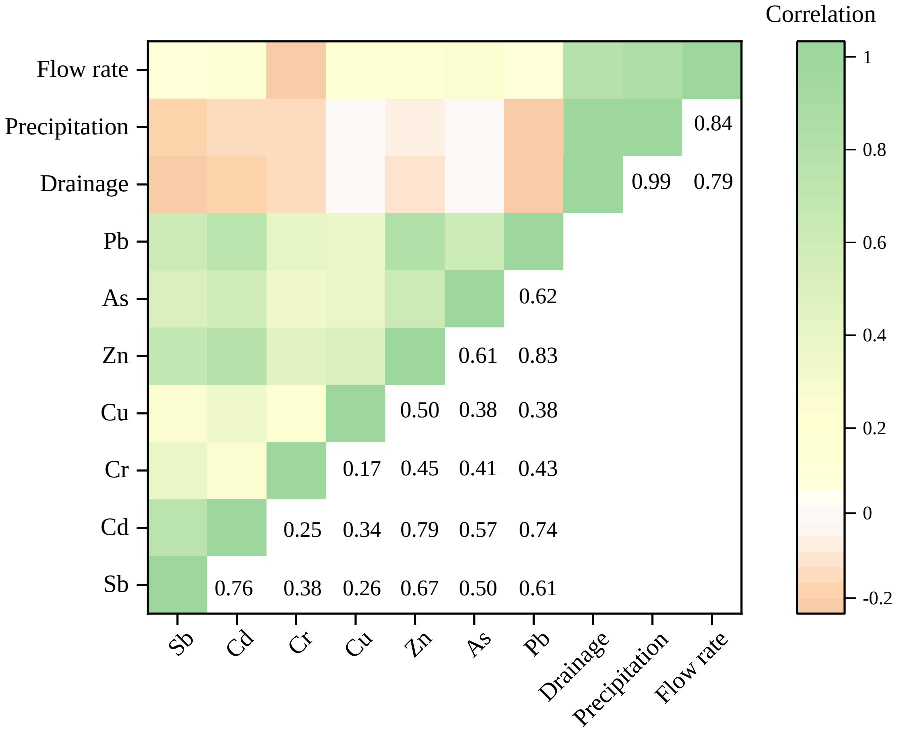

Figure S5. Pearson correlations among target metal elements and hydrological conditions in the Taipu River.

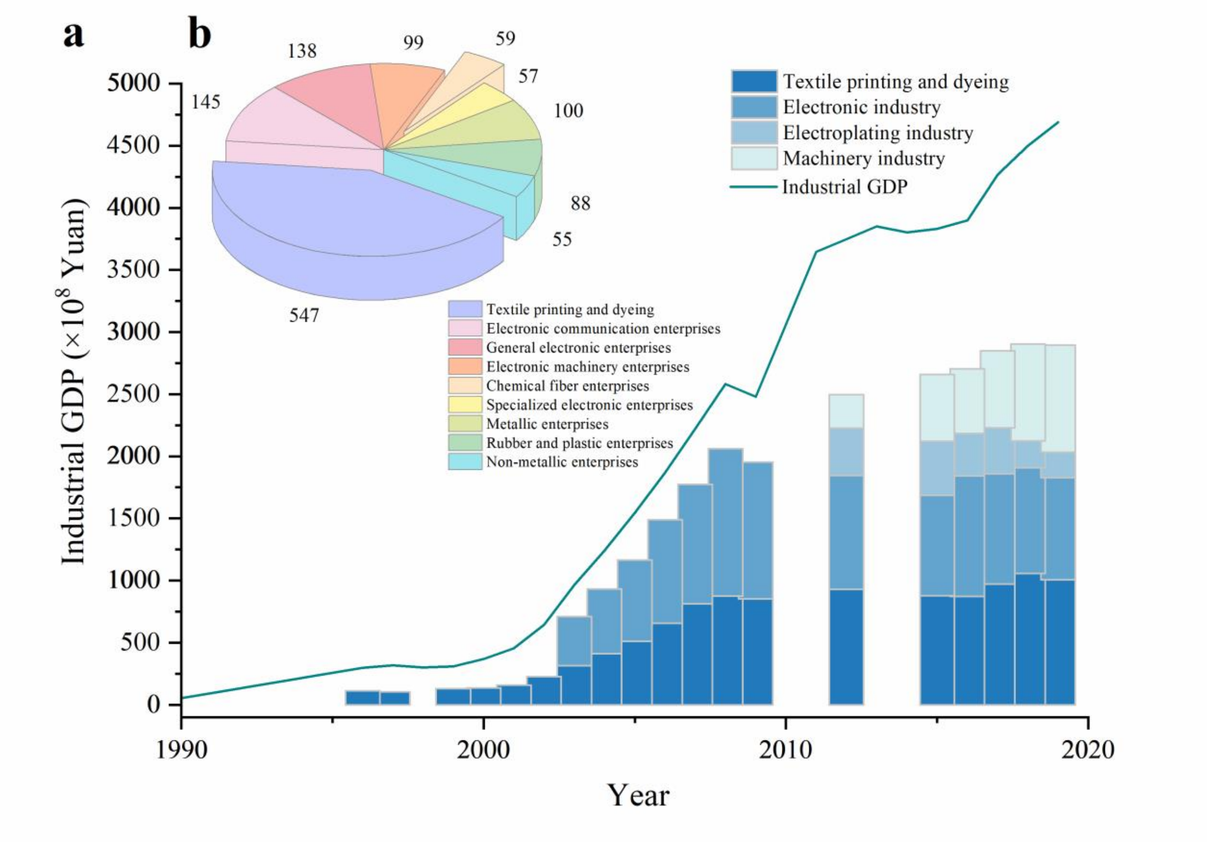

Figure S6. (a) Temporal trends of industrial and major industrial output values in Wujiang from 1990 to 2019 (b) Distribution of industrial enterprise types in Wujiang.

References

1. Tan, M. G.; Zhang, G. L.; Li, X. L.; Zhang, Y. X.; Yue, W. S.; Chen, J. M.; Wang, Y. S.; Li, A. G.; Li, Y.; Zhang, Y. M.; Shan, Z. C., Comprehensive study of lead pollution in Shanghai by multiple techniques. *Anal. Chem.* **2006,** *78* (23), 8044-8050.
2. Zheng, J.; Tan, M. G.; Shibata, Y.; Tanaka, A.; Li, Y.; Zhang, G. L.; Zhang, Y. M.; Shan, Z., Characteristics of lead isotope ratios and elemental concentrations in PM10 fraction of airborne particulate matter in Shanghai after the phase-out of leaded gasoline. *Atmospheric Environment* **2004,** *38* (8), 1191-1200.
3. Chen, J. M.; Tan, M. G.; Li, Y. L.; Zhang, Y. M.; Lu, W. W.; Tong, Y. P.; Zhang, G. L.; Li, Y., A lead isotope record of shanghai atmospheric lead emissions in total suspended particles during the period of phasing out of leaded gasoline. *Atmospheric Environment* **2005,** *39* (7), 1245-1253.
4. Bi, X.; Li, Z.; Wang, S.; Zhang, L.; Xu, R.; Liu, J.; Yang, H.; Guo, M., Lead Isotopic Compositions of Selected Coals, Pb/Zn Ores and Fuels in China and the Application for Source Tracing. *Environ. Sci. Technol.* **2017,** *51* (22), 13502-13508.
5. Zhu, B. Q.; Chen, Y. W.; Peng, J. H., Lead isotope geochemistry of the urban environment in the Pearl River Delta. *Appl. Geochem.* **2001,** *16* (4), 409-417.
6. Choi, M.; Yi, H.; Yang, S. Y.; Lee, C.; Cha, H., Identification of Pb sources in Yellow Sea sediments using stable Pb isotope ratios. *Mar. Chem.* **2007,** *107* (2), 255-274.
